# The pearl earrings

**DOI:** 10.1017/S1478951526102867

**Published:** 2026-06-02

**Authors:** Miguel Julião

**Affiliations:** Equipa Comunitária de Suporte em Cuidados Paliativos PalCo, ULS Amadora/Sintra, Amadora, Portugal

before you left,
I stood by the doorway of our place
and saw you so close
I could have whispered in your ear
how much I miss you―
even when I could still hold your hand.

before you left,
I stole from you―not a kiss,
for that was not possible―
but your pearl earrings,
the ones dropping softly by your neck.

I remember the bare nape of you,
your hair drawn up,
your perfume drifting toward me.

your pearl earrings―
pure, unblemished white―
rest now in the palm of my hand.

and I look at them so often,
standing by the door
where I wait to leave and find you,
or to see you return―

lifting your hair,
your silky nape laid bare,
your scent rising toward me,
asking me, using your eyes,
to place the pearl earrings
back upon your ears―

for only then, in that quiet union
of your skin, your hair, your perfume,
do they become what they are:
white as snow.

and then you smile―
and only I understand
the corners of your lips―
and you turn,
and move,
and begin to dance
like only you know.

And our place remembers its light―
for it shines again,
gently, quietly,
through your face.

